# Unusual Synchronous Colonic Metastasis of Ovarian Cancer

**DOI:** 10.7759/cureus.39952

**Published:** 2023-06-04

**Authors:** Laila Jaouani, Adil Zaimi, Ouissam Al Jarroudi, Anass Haloui, Fatima Rezzoug, Sami Aziz Brahmi, Said Afqir

**Affiliations:** 1 Department of Medical Oncology, Faculty of Medicine and Pharmacy, Centre Hospitalier Universitaire (CHU) Mohammed VI, Oujda, MAR; 2 Department of Medical Oncology, Faculty of Medicine and Pharmacy, Mohammed First University, Oujda, MAR; 3 Department of Medical Oncology, Mohammed VI University Hospital, Oujda, MAR; 4 Department of Pathology, Mohammed VI University Hospital, Oujda, MAR; 5 Department of Pathology, Faculty of Medicine, Mohammed First University, Oujda, MAR

**Keywords:** immunohistochemical, pegylated liposomal doxorubicin, platinum-sensitive disease, biomarker cancer antigen 125 (ca125), metastatic ovarian cancer, synchronous tumors

## Abstract

Colorectal metastasis is rare and can be confused with primary colorectal cancer. We report the case of a 63-year-old patient who presented with synchronous metastasis of the rectosigmoid junction and ovarian cancer. Initially thought to be a Krukenberg tumor, the diagnosis of metastasis from ovarian origin was confirmed through an immunohistochemical study of the colonic biopsy.

## Introduction

In Morocco, ovarian cancer is a significant public health problem, ranking third among female cancers [1]. In more than 35% of cases, it is only detected at stage IV of the disease. Its incidence is estimated between 2.7 and 3.1 per 100,000 inhabitants (Rabat Cancer Registry). Epithelial tumors account for 90% of ovarian cancers, and their dissemination occurs through four pathways: peritoneal, direct, lymphatic, and hematogenous [2]. Most often, metastases affect the liver, lungs, and brain [3]. We report the case of a patient who presented with both ovarian and colonic neoplasia simultaneously, posing a significant management challenge.

## Case presentation

**Figure 1:**
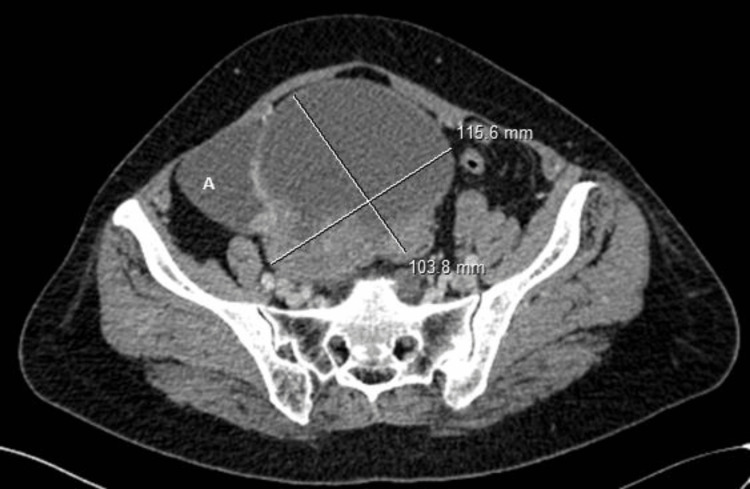
Abdominal computed tomography showing a solid-cystic pelvic mass with ascites (A) A: ascites

**Figure 2:**
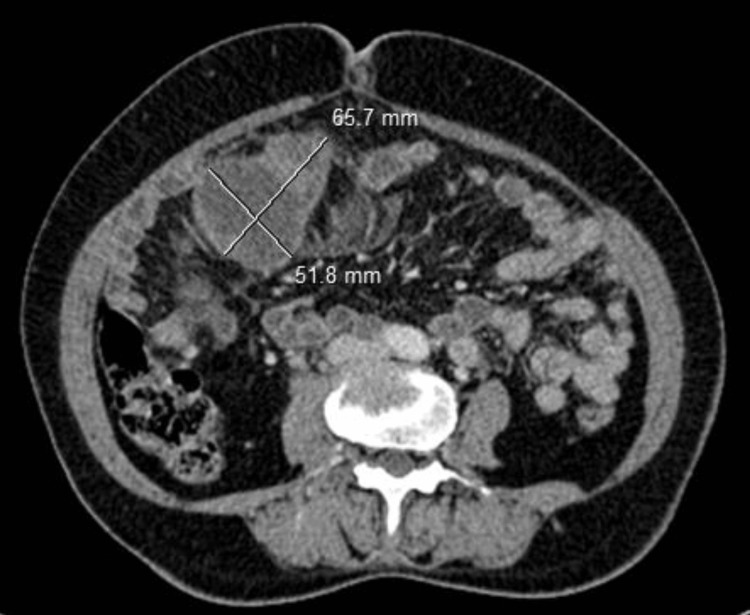
Solid-cystic peritoneal mass

**Figure 3:**
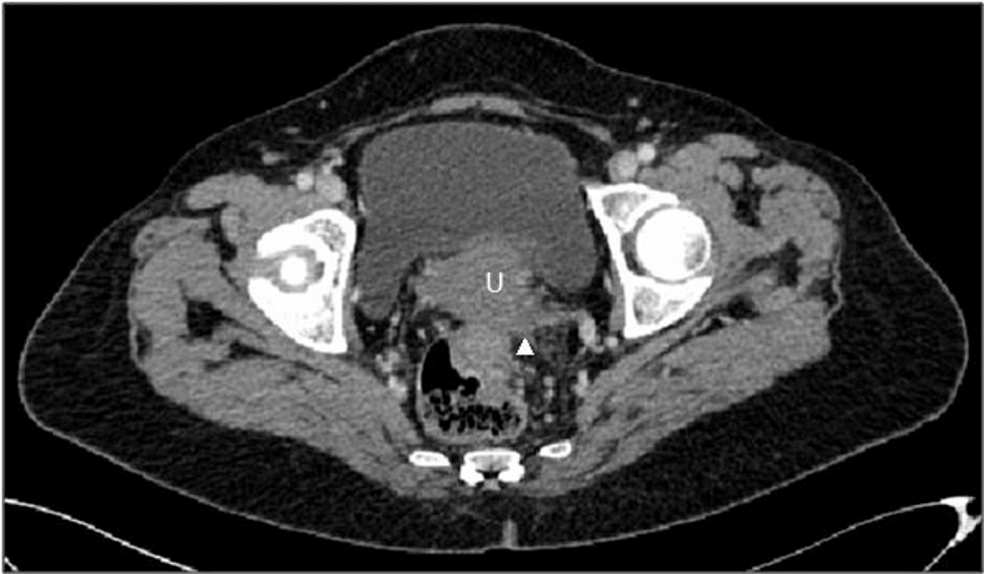
Tumoral thickening at the rectosigmoid junction (arrow) U: uterus

**Figure 4:**
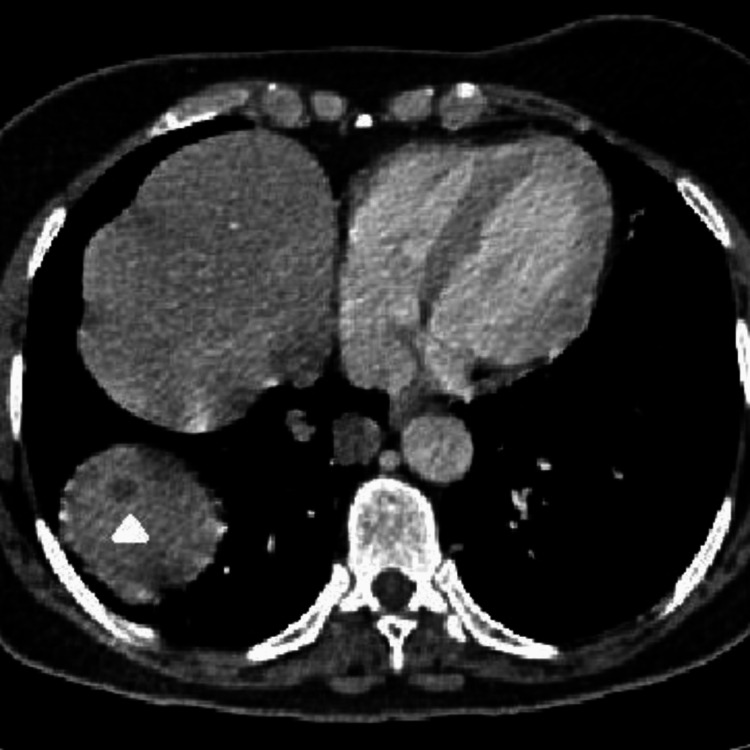
Hepatic metastasis (white triangle)

**Figure 5:**
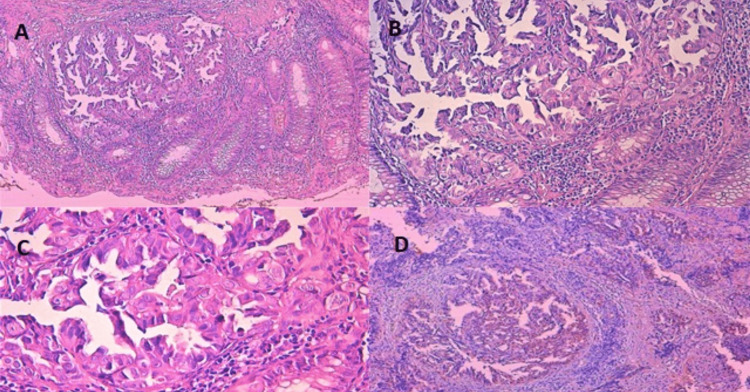
Pathological and immunohistochemical findings of rectal biopsy tissue A: Rectal mucosa infiltrated by a carcinomatous tumoral proliferation, displaying a papillary architecture and dissociating the muscularis mucosae. B: The papillae are endowed with discrete fibrovascular axes, lined with frankly atypical tumor cells. C: Tumor cells have irregular nuclei, sometimes hyperchromatic, sometimes with fine chromatin with a discrete nucleolus. The cytoplasm is abundant and eosinophilic. A few mitotic figures can be seen. D: Nuclear expression of WT1 by tumor cells. WT1: Wilms’ tumor 1

The patient was a 63-year-old female with no previous medical history who presented to the emergency department with pelvic pain and abdominal distension for a duration of two months. A cervico-thoraco-abdomino-pelvic computed tomography (CT) scan revealed a solid-cystic pelvic mass measuring 115 × 103 mm (Figure 1), a peritoneal mass (Figure 2), and minimal ascites, as well as thickening of the rectosigmoid junction suggestive of a tumor (Figure 3). Hepatic and pulmonary metastases were also found (Figure 4), and a Krukenberg tumor associated with peritoneal carcinomatosis was suspected. The cancer antigen 125 (CA125) marker was significantly elevated at 1,316 UI/mL, while carcinoembryonic antigen (CEA) was negative at 1 ng/mL. The upper gastrointestinal endoscopy was normal, and the rectosigmoidoscopy revealed an ulcerating and budding process located 15 cm from the anal margin. The biopsy with histopathological examination was in favor of a well-differentiated adenocarcinoma infiltrating the chorion with foci of tumor necrosis (Figure 5).

The case was discussed at a multidisciplinary meeting, and exploratory laparotomy with surgical resections was indicated. The surgery consisted of left adnexectomy, right annexial cytoreduction, hepatic biopsy, and resection of the peritoneal mass. Histopathology revealed a bilateral high-grade serous cystadenocarcinoma of the ovaries with hepatic metastasis and peritoneal carcinomatosis and immunohistochemical labeling results (cytokeratin 20 (CK20) negative, caudal-type homeobox protein 2 (CDX2) negative, human Wilms’ tumor 1 (WT1) gene positive, inhibin negative, and calretinin negative). Therefore, the patient was diagnosed with stage IV metastatic serous cystadenocarcinoma of the ovary (with metastases to the liver, lung, and peritoneum) associated synchronously with an adenocarcinoma of the rectosigmoid junction.

Palliative chemotherapy was proposed in a multidisciplinary meeting, followed by digestive surgery in case of a favorable response. The patient received carboplatin AUC 5 + paclitaxel 175 mg/m^2^ + bevacizumab 7.5 mg/kg every 21 days for three cycles, and a partial response to treatment was obtained at this time. However, a clear radiological and biological progression was observed after the completion of six cycles: CA125 at 118 UI/mL versus 16.6 UI/mL, CEA still negative at 1.09 ng/mL, and a significant increase in the size of liver and lung metastases at CT evaluation.

**Figure 6:**
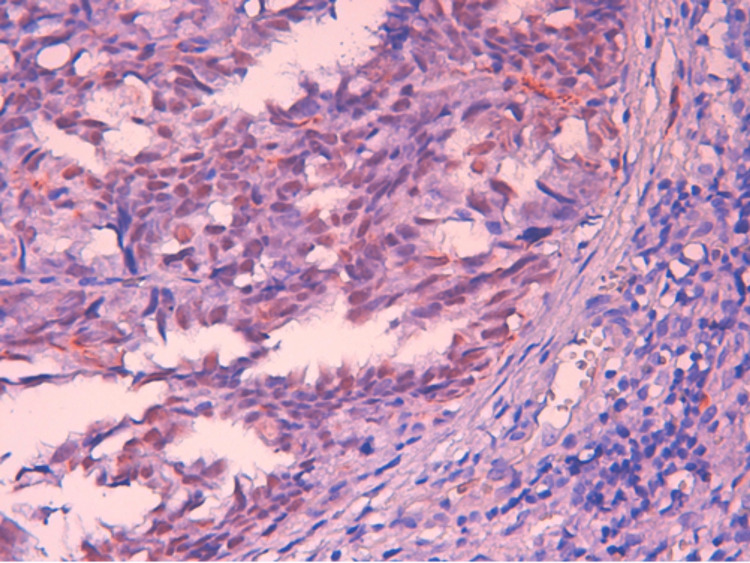
Nuclear expression of WT1 by tumor cells (higher magnification) WT1: Wilms’ tumor 1

The case was reconsidered in the multidisciplinary meeting consultation, and the decision was to continue the same chemotherapy protocol given the initial good response. The patient received an additional six cycles, for a total of 12, with a favorable evolution of the ovarian, peritoneal, rectosigmoid, hepatic, and pulmonary regions and normalization of tumor markers (CA125 at 10 UI/mL and carcinoembryonic antigen (CEA) remained negative at 0.91 ng/mL). Given the clear clinical, biological, and radiological benefits, an additional immunohistochemical study of the rectal biopsy was performed. The result suggested a gynecological origin, notably ovarian adenocarcinoma: CK7 positive, WT1 positive, CK20 negative, and CDX2 negative (Figure 6). The patient was placed on therapeutic pause with stable disease.

Eight months later, an increase in CA125 to 360 UI/mL with progression of the tumor was noted. Palliative chemotherapy was resumed with carboplatin AUC 5 + gemcitabine 1,000 mg/m^2^ + bevacizumab 7.5 mg/kg for a total of six cycles with good clinical evolution, a CA125 of 92 UI/mL, and radiological stability. Maintenance treatment with bevacizumab was given.

Six months later, a biological and radiological progression was observed. Several lines of palliative chemotherapy were initiated, without significant benefit. The patient survived for five years from diagnosis.

## Discussion

Colonic metastasis of ovarian cancer is very rare. In our case, it can be confused with primary colorectal cancer. Four routes of metastatic dissemination of ovarian cancer have been described. It can involve a direct extension to neighboring organs and peritoneal diffusion or intraluminal/intramural involvement via hematogenous or lymphatic spread; colonic involvement occurs through invasion of the serosa and wall in a centripetal fashion [4]. Reed et al. [5] identified peritoneal involvement in 83%-100% and colon involvement in 50%-60% of patients with advanced ovarian cancer in an autopsy study. Our case is unusual given that parietal involvement is confined to the mucosa and chorion, suggesting a hematogenous or lymphatic route. Reed et al. [5] described infiltration of the submucosal capillary network in rectocolic metastases.

Colonic metastasis via a hematogenous route from ovarian cancer is rare. It has also been reported in breast, lung, and melanoma cancers [6,7]. The presence of synchronous metastasis from an evolving ovarian cancer is even rarer [8]. Loy et al. [9] reported seven cases in a series.

The rectosigmoid and left colon are the most frequent sites of metastasis originating from ovarian cancer [10]. The time interval from the initial diagnosis of ovarian cancer to the appearance of metastasis varies widely, ranging from one to 22 years [6].

The clinical presentation is comparable to that of primary colorectal tumors. It can manifest as intestinal obstruction, lower gastrointestinal bleeding, perforation, or anemia. Asymptomatic cases may delay the diagnosis.

The workup includes an abdominopelvic CT scan or ultrasound, which may reveal a unilateral or bilateral pelvic mass, colorectal lymphadenopathy, ascites, or colonic wall thickening [11]. Colonic endoscopy with biopsy and histopathological examination is necessary for the diagnosis and identification of the primary tumor [11,12]. Immunohistochemical analysis of CK7 and CK20 is essential to differentiate primary colorectal cancer from metastasis. Tumor markers such as CEA, CA125, and carbohydrate antigen 19-9 (CA19-9) play an additional role in distinguishing primary colorectal cancer from ovarian cancer metastasis and evaluating treatment response [13,14]. The positivity of CK7 and CA125 indicates ovarian origin, while that of CK20 and ACE suggests colorectal origin. It should be noted that CA125 remains negative in 15% of ovarian cancers [15]. CK7-positive/CK20-negative immunophenotype is specific for ovarian origin, while CK7-negative/CK20-positive phenotype strongly suggests colorectal origin [16,17]. Unlike secondary colorectal cancer, secondary ovarian cancer is common. The immunohistochemical marker WT1 is useful for distinguishing secondary ovarian cancer from primary cancer. It is positive in the majority of serous ovarian and peritoneal carcinomas, mesotheliomas, and Wilms’ tumors [18].

In our case, a colorectal tumor presenting CK7 positive/CK20 negative and WT1 positive is compatible with an ovarian origin. The management is done in a multidisciplinary consultation meeting including the gynecologic surgeon, pathologist, oncologist, and radiologist. According to European recommendations [19], the standard therapy consists of a combination of carboplatin AUC 5 and paclitaxel 175 mg/m^2^ administered intravenously every three weeks. This association has been adopted for over 15 years [20]. Other alternatives have been proposed with other combinations that have a confirmed similar efficacy through randomized clinical trials, notably pegylated liposomal doxorubicin (PLD)-carboplatin and docetaxel-carboplatin.

The addition of bevacizumab at a dose of 15 mg/kg, which is a monoclonal antibody targeting vascular endothelial growth factor receptor (VEGFR), has improved overall survival and progression-free survival for definitively non-resectable stages IIIC-IV in the International Collaborative Ovarian Neoplasm (ICON) 7 trial [21]. The principle of maintenance therapy after first-line treatment is often discussed in ovarian cancer due to the clear benefit in terms of progression-free survival of 19.6 months, especially with niraparib and bevacizumab, even in the absence of germline BRCA1 or BRCA2 mutations. In our context, maintenance therapy was provided by bevacizumab with a satisfactory therapeutic response.

Our patient had partial sensitivity to platinum salt. The preferred treatment was the combination of carboplatin and gemcitabine. However, the tumor later became refractory, which predicts a reduced overall survival and poor prognosis. At this stage, our patient was administered four agents (weekly paclitaxel, topotecan, pegylated liposomal doxorubicin (PLD), and gemcitabine), which have shown some effectiveness in phase III trials [22]. Our patient had an overall survival of five years, which can be considered satisfactory for stage IV ovarian disease.

## Conclusions

The significance of this clinical case is to emphasize the possibility of hematogenous spread of ovarian cancer to the colon. It represents a real diagnostic challenge, especially in cases of synchronicity. The crucial role of immunohistochemistry in differential diagnosis has to be highlighted.

Although metastatic ovarian cancer remains a poor prognosis, new modalities have clearly increased progression-free survival in a clinically and statistically significant way, and our patient confirms it.
